# Use of portable single‐lead electrocardiogram device as an alternative for QTc monitoring in critically ill patients

**DOI:** 10.1111/anec.13116

**Published:** 2024-04-16

**Authors:** Martin Rebolledo‐Del Toro, Ana Beatriz Carvajalino‐Galeano, Clarena Pinto‐Brito, Oscar Mauricio Muñoz‐Velandia, Ángel Alberto García‐Peña

**Affiliations:** ^1^ Division of Cardiology Hospital Universitario San Ignacio Bogota Colombia; ^2^ Department of Internal Medicine Pontificia Universidad Javeriana Bogota Colombia; ^3^ Department of Medicine Pontificia Universidad Javeriana Bogota Colombia; ^4^ Department of Internal Medicine Hospital Universitario San Ignacio Bogota Colombia

**Keywords:** electrocardiography, KardiaMobile, mobile applications, QT/QTc interval, single‐lead ECG device

## Abstract

**Purpose:**

Acquired QT prolongation is frequent and leads to a higher mortality rate in critically ill patients. KardiaMobile 1L® (KM1L) is a portable, user‐friendly single lead, mobile alternative to conventional 12‐lead electrocardiogram (12‐L ECG) that could be more readily available, potentially facilitating more frequent QTc assessments in intensive care units (ICU); however, there is currently no evidence to validate this potential use.

**Methods:**

We conducted a prospective diagnostic test study comparing QT interval measurement using KM1L with conventional 12‐L ECG ordered for any reason in patients admitted to an ICU. We compared the mean difference using a paired *t*‐test, agreement using Bland–Altman analysis, and Lin's concordance coefficient, numerical precision (proportion of QT measurements with <10 ms difference between KM1L and conventional 12‐L ECG), and clinical precision (concordance for adequate discrimination of prolonged QTc).

**Results:**

We included 114 patients (61.4% men, 60% cardiovascular etiology of hospitalization) with 131 12‐L ECG traces. We found no statistical difference between corrected QT measurements (427 ms vs. 428 ms, *p* = .308). Lin's concordance coefficient was 0.848 (95% CI 0.801–0.894, *p* = .001). Clinical precision was excellent in males and substantial in females (Kappa 0.837 and 0.781, respectively). Numerical precision was lower in patients with vasoactive drugs (−13.99 ms), QT‐prolonging drugs (13.84 ms), antiarrhythmic drugs (−12.87 ms), and a heart rate (HR) difference of ≥5 beats per minute (bpm) between devices (−11.26 ms).

**Conclusion:**

Our study validates the clinical viability of KM1L, a single‐lead mobile ECG device, for identifying prolonged QT intervals in ICU patients. Caution is warranted in patients with certain medical conditions that may affect numerical precision.

## Graphical abstract

This study validates the clinical precision of KM1L, a single‐lead mobile ECG device, for identifying prolonged QT intervals in ICU patients. Caution is warranted in certain medical conditions that may affect numerical precision.

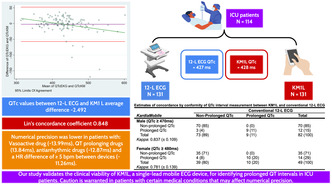

## 1 INTRODUCTION

Cardiovascular diseases, including malignant arrhythmias, are among the leading causes of mortality and intensive care unit (ICU) admissions (Reinelt et al., 2001; World Health Organization, 2021). Critically ill patients frequently exhibit acquired QT interval prolongation attributed to multiple risk factors, such as electrolyte imbalances, renal dysfunction, and the administration of QT‐prolonging medications (Armahizer et al., 2013; Beitland et al., 2014; Gorard, 2006; Papukhyan et al., 2020; Pickham et al., 2012; Tisdale et al., 2012). Acquired long QT syndrome, particularly exceeding 500 ms, correlates with an elevated risk of ventricular arrhythmias, sudden cardiac death, and both short‐ and long‐term mortality (George et al., 2015; Javanainen et al., 2019; Kallergis et al., 2012; Pickham et al., 2012).

Traditionally, the 12‐lead electrocardiogram (12‐L ECG) has served as the benchmark for QT interval assessment, requiring heart rate correction for accuracy (Indraratna et al., 2020; Papukhyan et al., 2020). Clinical demands in the ICU may require rigorous QT interval monitoring, which can be difficult with conventional 12‐L ECG due to logistical limitations. The possibility of frequent QTc monitoring, with minimal contact from healthcare personnel proves advantageous, notably in infectious environments (Asensio et al., 2020). Telemetry monitors with real‐time QTc measurement exhibit poor concordance and only moderate correlation, with one third of patients exhibiting different measurements between telemetry and 12‐L ECG, particularly patients with baseline ECG abnormalities, which are not infrequent in ICU setting (Nasser et al., 2021; Papukhyan et al., 2020). While continuous cardiac monitoring is standard in many ICUs, this is not universally true. Resource constraints in some hospitals, particularly in developing countries, can limit access to such technology. Even where available, systems may not focus on QTc, potentially delaying interventions. Contemporary advances in mobile technology offer versatile solutions for remote monitoring beyond traditional medical devices. KardiaMobile 1L® (KM1L) is a commercially available, user‐friendly, and relatively inexpensive technological innovation that enables quick acquisition of a single‐lead electrocardiographic tracing within 30 s (AliveCor, 2021; AliveCor Inc, 2017; Haberman et al., 2015). Previous research has demonstrated the utility of this tool in detecting arrhythmias, assessing conduction system abnormalities, monitoring QT/QTc intervals, and diagnosing myocardial infarction in outpatient settings (Chung & Guise, 2015; Muhlestein et al., 2015, 2018; Treskes et al., 2017; Veale et al., 2018). Obtaining a 12‐lead ECG typically involves additional nursing staff being exposed to equipment fitted with numerous ECG wires, heightening the risk of contagious disease transmission. KM1L offers several benefits, including ease of use (patients can be instructed to operate it, reducing contact with healthcare personnel), remote data transmission (thus reducing the risk of contamination), compact size, and easy disinfection (Alshamrani et al., 2022; Chu et al., 2020; González et al., 2020; Klompas et al., 2021; Zaballos et al., 2023). In hospitalized patients, comparisons with a standard 12‐L ECG demonstrate the KM1L device's noteworthy numerical accuracy and exceptional agreement in identifying QTc prolongation (Marín et al., 2021). Nevertheless, existing literature lacks validation regarding the utility of KML1 QT measurement within the critical care population (Bansal & Joshi, 2018).

Given the high mortality rates associated with QT interval prolongation, particularly in critically ill patients, it is essential to find substitutes for the traditional 12‐L ECG for regular monitoring of QT intervals. This will help to reduce the risks of spreading infections among healthcare personnel, identify individuals who are at greater risk of QTc prolongation, and provide early management of medications that have the potential to prolong QT intervals. Such a pragmatic and cost‐effective approach can help to mitigate in‐hospital mortality (Tisdale et al., 2012). The use of a KM1L device for QT measurements offers a more expeditious and user‐friendly alternative to conventional ECGs, potentially facilitating more frequent QTc assessments in critically ill patients. This study compares the clinical precision and concordance of QTc interval measurements obtained through 12‐L ECGs with those derived from a single lead using KM1L.

## 2 MATERIALS AND METHODS

This was a prospective diagnostic test study aimed at assessing accuracy, precision, and concordance of a portable single‐lead electrocardiographic device compared with the 12‐L ECG to identify QT prolongation, conducted at a reference hospital in Bogotá, D.C., Colombia. The population included a consecutive sample of critically ill patients aged 18 years or older, admitted to the ICU at the *Hospital Universitario San Ignacio* from July 2022 to May 2023. Exclusion criteria comprised patients with atrial fibrillation, active external electrical stimulation, and individuals with hand amputations (necessary for use of KM1L). We received ethical approval from Local Ethics Committee (FM‐CIE‐0341‐22, 06/2022). The study was conducted following standards specified in the International Council for Harmonization Guidelines for Good Clinical Practice and in adherence to the principles of the Declaration of Helsinki.

For each patient, both a conventional 12‐L ECG and a KM1L tracing were consecutively performed. More than one 12‐L ECG and KM1L tracing could be obtained for a single patient if the attending physician considered ordering another during the ICU stay. All clinical decisions were made using the conventional 12‐L ECG trace only.

KM1L, a portable single‐lead electrocardiographic mobile device, was utilized. This device is compatible with iOS and Android platforms, connects wirelessly to smartphones, and consists of two stainless steel electrodes that contact the patient's fingers to record a bipolar lead. The software application used for recording and analysis is called Kardia® (AliveCor, San Francisco, CA). To record with the KM1L, one finger from each hand was placed on the device's electrodes for at least 30 s. Whenever possible, the patient was handed the device and instructed to do this on his own, minimizing contact with the nurse personnel. An electrocardiographic trace in DI is generated within the Kardia app, saved in PDF format, labeled, and sent via email for analysis. In the conventional 12‐L ECG, leads DII and V5 were used to manually measure QT intervals. QTc values were calculated using Bazett's formula, considering three consecutive QTs and their corresponding R‐R intervals. These measurements were conducted by three expert investigators who performed blind measurements without knowledge of the results of the other test and defined QT and QTc values through consensus.

The general characteristics of the study population were analyzed using descriptive statistics. Absolute and relative frequencies were calculated for qualitative variables. For quantitative variables, measures of central tendency (mean and standard deviation for normally distributed data or median and interquartile range for non‐normally distributed data) were calculated.

The difference between QTc values was analyzed using a paired *t*‐test to determine the mean difference between measurements obtained from the conventional 12‐L ECG and the portable ECG device. Bland–Altman analysis and Lin's concordance coefficient were used to test the agreement between QTc measured with both devices. Numerical precision was defined as the proportion of QT and QTc measurements in which the KM1L value had a difference of less than 10 ms compared to conventional 12‐L ECG values. Clinical precision was assessed by examining concordance by conformity between QTc values measured by the KM1L and conventional 12‐L ECG, with the conventional 12‐L ECG considered the reference standard. For each measurement method, two categories were established based on the presence or absence of QTc interval prolongation, with cutoff values of ≥470 ms for postpubertal males and ≥480 ms for postpubertal females. Concordance between diagnostic methods was assessed using kappa statistics with a significance level of alpha at 0.05.

A subanalysis was performed, to consider the subpopulation of patients with risk factors for QTc prolongation. A backward stepwise linear regression model was used to identify the reasons for the differences in measurements between the two devices. The model aimed to assess the magnitude of the effect of each variable on the differences in QTc measurements, thus allowing quantification of their impact on the observed variations.

The sample size calculation was based on a paired means difference with a 10 ms difference between methods, assuming values of 450 ms and 460 ms, a standard deviation of 40 ms, a 10% attrition rate, a type I error of 0.05, and a type II error of 20%. This estimation yielded a total of 256 ECG traces, with 128 measurements for each method. Statistical significance was considered at *p* < .05, with 95% confidence intervals. Data processing and statistical analyses were carried out using STATA 16 (StataCorp LLC, College Station, TX) software.

## 3 RESULTS

### 3.1 Baseline characteristics

One hundred and fourteen patients were included, 61.4% of them were men, with an age median of 64 years. The main indication for hospitalization was for cardiovascular disease in 60% of patients. More than half of patients had a history of heart failure, with ischemic etiology being the most frequent. One fifth had invasive mechanical ventilation and vasoactive support. One‐third had antiarrhythmic medication; potentially QT‐prolonging drugs were present in 32.5% of patients; and 41.2% had electrolyte disturbance. Table 1 presents other baseline characteristics.

**TABLE 1:** Patient baseline characteristics.

Characteristics	Results (*n* = 114)
Men, *n* (%)	70 (61.4)
Age in years, median (IQR)	64 (52–73)
Main indication of hospitalization in ICU, *n* (%)	
Cardiovascular	69 (60.5)
Noncardiovascular	45 (39.5)
# Hospital days, median (IQR)	12 (7–20)
# ICU days, median (IQR)	5 (3–8)
Invasive mechanical ventilation, *n* (%)	23 (20.4)
History of heart failure, *n* (%)	68 (59.6)
Ischemic	57 (50.4)
Valvular	12 (10.5)
Other	14 (12.3)
History of hypertension, *n* (%)	75 (66.4)
History of diabetes mellitus, *n* (%)	45 (39.5)
Antiarrhythmic medication, *n* (%)	38 (33.3)
Amiodarone	8 (7.1)
Beta blocker	35 (30.7)
Lidocaine	4 (3.5)
Other	3 (2.6)
Vasoactive medication, *n* (%)	23 (20.2)
Noradrenaline	15 (13.2)
Dobutamine	13 (11.4)
Vasopressin	4 (3.5)
Levosimendan	3 (2.6)
Milrinone	1 (0.9)
Other	5 (4.4)
Antimicrobian, *n* (%)	22 (19.3)
Macrolide	1 (0.9)
Azole antifungal	3 (2.6)
TMP/SMX	1 (0.9)
ART	2 (1.8)
Other	20 (17.5)
Other potentially QT‐prolonging drugs, *n* (%)	37 (32.5)
Haloperidol	4 (3.5)
Quetiapine	1 (0.9)
Ondansetron	3 (2.6)
P2Y12 inhibitor	25 (21.9)
Hydroxychloroquine	1 (0.9)
Steroid	7 (6.1)
Benzodiazepine	5 (4.4)
Levetiracetam	4 (3.5)
Vecuronium	2 (1.8)
Electrolyte disturbance, *n* (%)	47 (41.2)
Hypokalemia	28 (24.6)
Hypomagnesemia	24 (21.2)
Hypocalcemia	5 (4.39)

Abbreviations: ART, antiretroviral therapy; ICU, intensive care unit; IQR, interquartile range; *n*, number; TMP/SMX, trimethoprim/sulfamethoxazole.

### 3.2 QT interval averages

One hundred and thirty‐one 12‐L ECG and KM1L traces were collected for analysis. The median measured QT was 400 ms (IQR 360–430 ms) for 12‐ L ECG and 400 ms (IQR 350–430 ms) for KM1L, with no statistical difference between them (*p* = .7457). The corrected QT measurement median and IQR were 427 ms (405–456 ms) for 12‐L ECG and 428 ms (399–459 ms) for KM1L, with no statistical difference between them (*p* = .308).

### 3.3 Agreement between conventional 12‐L ECG and portable ECG device

**FIGURE 1:**
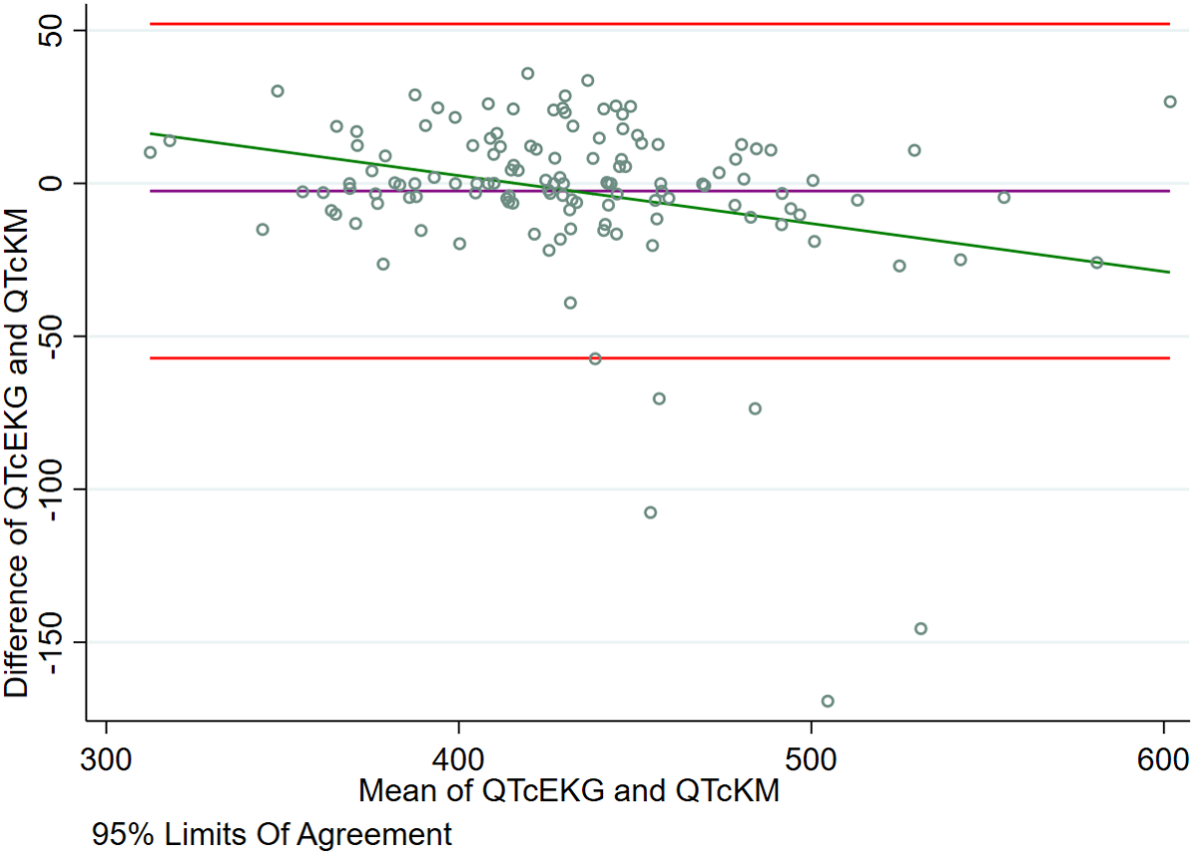
Bland‐Altman analysis in QTc values bewtween conventional 12‐L ECG and portable ECG device KM1L. Abbreviations: 12‐L ECG, 12 lead electrocardiogram; KM1L, KardiaMobile 1L; QTc, corrected QT.

The Bland–Altman analysis (Figure 1) in QTc values between conventional 12‐L ECG and portable ECG devices showed an average difference of −2.492 (95% limits of agreement: −57.109; 52.125) (Slope 0.864). Lin's concordance coefficient was 0.848 (95% CI 0.801–0.894, *p* = .001). In the lower range of QTc, values were underestimated by KM1L, while in higher QTc values, KM1L measurement led to overestimation.

### 3.4 Concordance by conformity

Concordance between the KM1L device and the conventional 12‐L ECG to determine the presence of QTc prolongation is shown in Table 2. Prolongation corresponded to a QTc value of ≥480 ms in female patients and ≥470 ms in male patients. Concordance was excellent in males and substantial in females (kappa 0.837 and 0.781, respectively).

**TABLE 2:** Estimates of concordance by conformity of QTc interval measurement between KM1L and conventional 12‐L ECG.

*KardiaMobile*	Conventional 12‐L ECG		
	Nonprolonged QTc	Prolonged QTc	Total
Male (QTc ≥ 470 ms)			
Nonprolonged QTc	70 (85)	0 (0)	70 (85)
Prolonged QTc	3 (4)	9 (11)	12 (15)
Total	73 (89)	9 (11)	82 (100)
*Kappa*: 0.837 (±0.109)			
Female (QTc ≥ 480 ms)			
Nonprolonged QTc	35 (71)	0 (0)	35 (71)
Prolonged QTc	4 (8)	10 (20)	14 (29)
Total	39 (80)	10 (20)	49 (100)
*Kappa*: 0.781 (±0.139)			

*Note*: Data are shown by number (% of total patients in the group). Kappa value ± standard error is also shown.

Abbreviations: 12‐L ECG, 12 lead electrocardiogram; KM1L, KardiaMobile 1L®; QTc, corrected QT.

### 3.5 Numerical precision

Numerical precision, defined as the proportion of measurements in which QT interval measurement from KM1L had less than 10 ms of difference with the measurement in conventional 12‐L ECG, was 48.9% for QTc. Three traces of 2 patients had extreme discrepancies, with differences greater than 100 ms. The first patient had a marked repolarization abnormality with a biphasic T wave and a suboptimal KM1L tracing. The second patient had an 18 ‐bpm difference between the 12‐L ECG and KM1L measurements.

### 3.6 Subgroup

A subgroup analysis of numeric precision was conducted with different characteristics shown in Table 3. A heart rate (HR) difference between measurements in KM1L and 12‐L ECG of ≥5 bpm led to a significant statistical difference in numerical precision (28.1% vs. 55.6%, *p* = .007). A 5 bpm difference led to a significant change of 11 ms in QTc between methods. Although nonstatistically significant, a numerical difference was also found in the presence of vasoactive medication and hypokalemia.

**TABLE 3:** Sub‐group analysis in function of KM1L numeric precision compared to conventional 12‐L ECG.

Characteristics, *n* = 131	Numerical precision^b^	*p*
Gender		
Male	51.2	.484
Female	44.9	
Invasive mechanical ventilation		
Yes	42.3	.380
No	52.0	
History of heart failure		
Yes	48.0	.517
No	53.9	
Abnormal HR		
Tachycardia	50.0	1.000
Bradycardia	48.2	.467
≥5 lpm difference in HR		
Yes	28.1	.007^c^
No	55.6	
≥1 QT altering factor^a^		
Yes	47.4	.699
No	50.9	
Any antiarrhythmic medication		
Yes	50.0	.897
No	48.8	
Any vasoactive medication *n* (%)		
Yes	33.3	.063
No	53.4	
Any antimicrobian		
Yes	46.2	.725
No	50.0	
Other potentially QT‐prolonging drugs		
Yes	48.8	.950
No	49.4	
Hypokalemia		
Yes	60.6	.118
No	44.9	
Hypomagnesemia		
Yes	55.6	.434
No	47.1	

*Note*: Data are presented as percentage. *p* Value obtained through *X* ^2^ test.

Abbreviations: 12‐L ECG, 12 lead electrocardiogram; HR, heart rate; KM1L, KardiaMobile 1L®.

^a^ QT altering factor includes: any antiarrhythmic, vasoactive, antimicrobial, other QT prolonging drugs, hypokalemia, or hypomagnesemia.

^b^ Numerical precision was defined as the proportion of KardiaMobile (Alive‐Cor, San Francisco, CA) QT interval measurements with less than 10 ms difference from conventional electrocardiogram measurements.

^c^ *p* < .05.

### 3.7 Regression model

A backward stepwise regression model was conducted in order to find the variables that could explain the difference between methods, as well as the size of the effect. Results are shown in Table S1. We found that the factors that significantly explained the differences were the presence of hypokalemia, vasoactive medication, antiarrhythmic drugs, other potentially QT prolonging drugs, and a HR difference of ≥5 bpm between measurements. The factors with the wider differences in QTc measurement between devices were the presence of vasoactive drugs (−13.99 ms), QT‐prolonging drugs (13.84 ms), antiarrhythmic drugs (−12.87 ms), and a HR difference of ≥5 bpm (−11.26 ms).

## 4 DISCUSSION

Our study evaluating the agreement of a portable 1‐lead ECG device compared with conventional 12‐L ECG in critically ill patients found no statistical difference in median QT and QTc measurements, with excellent concordance in men and substantial concordance in women for clinical discrimination of a prolonged QTc value. Nevertheless, agreement and numerical precision were poor. With the use of subgroup analysis and a linear regression model, we found that the presence of certain QT‐prolonging risk factors, as well as a difference of ≥5 bpm between HR measurements, led to a statistically significant difference in diagnostic performance.

This is not the first time the KM1L has been used to monitor QTc previous studies have shown a sensibility greater than 80% and a specificity of 94% for the detection of prolonged QT% (Himmelreich et al., 2019). In a study conducted by Beers et al. in 2021 evaluating the precision of QT measurement with KM1L compared with 12‐L ECG in 125 patients, they found no statistical difference in mean measurements between devices, with excellent agreement in 66.9% and clinically acceptable agreement in 93.4% of observations. This study, however, included only patients with any nonacute indication in primary care (Beers et al., 2021).

A more recent study by Marin et al. compared KM1L with conventional 12‐L ECG for measurement of QTc, specifically in patients hospitalized for suspected or confirmed COVID‐19. Even though not critically ill, about half of patients reported the use of medications with the potential to prolong the QTc interval. Like our findings, they reported no difference between the QTc interval measured with KM1L or conventional 12‐L ECG (442.5 ± 40.5 vs. 442.4 ± 40.2 ms, *p* = .986) with excellent agreement (Lin concordance coefficient 0.988, 95% CI: 0.983–0.992, *p* < .001). Clinical precision was excellent in both women (Kappa: 0.901) and men (Kappa: 0.896); however, there was a higher proportion of QTc prolongation for females in our study (20%). Numerical precision was higher than in our study, 93% for the whole population. They found lower concordance in patients with confirmed SARS‐CoV‐2 infection. They hypothesized that it could be explained by greater variation in heart rates associated with autonomic abnormalities, particularly when associated with a change in position from supine to sitting, which was sometimes needed in order to obtain an adequate quality KM1L trace. This study, however, excluded critically ill patients admitted to the ICU (Marín et al., 2021).

In 2023, Zaballos et al. published a feasibility study using a 6‐lead KardiaMobile (KM6L) for QT interval monitoring in critical care patients (Zaballos et al., 2023). They found no statistical differences in QTc measured by each method, with good agreement, however, there are some limitations to their findings: The study was conducted only in confirmed SARS‐CoV2‐infected patients, limiting the generalizability of the findings to other populations. The sample size was minimal, with only 20 patients included, reducing the confidence in the results. No analysis was made regarding cases in which the two devices differed from each other. Additionally, the KML6 requires the device to be placed on the patient's left thigh bare skin, which means greater complexity, more contact with the patient, and potentially longer exposure to healthcare personnel. Furthermore, the KM6L is almost twice as expensive and has no proven advantage in clinical applicability over the KM1L (AliveCor, Inc, 2023; The Skeptical Cardiologist, 2021).

Even though we found no difference in central tendency measurements between devices, along with excellent and substantial clinical precision, numerical precision was lower than in previous studies. There could be many possible explanations for this phenomenon that are supported by other findings from the study. We found that in the lower range of QTc, values were underestimated by KM1L, while in higher QTc values, KM1L measurement led to overestimation. Also, in subgroup analysis, we found that a heart rate difference between measurements in KM1L and 12‐L ECG of ≥5 bpm led to a significant statistical difference in numerical precision, with a significant change of 11 ms in QTc between methods. During the KM1L acquisition, a change in position from supine to sitting was often needed. One of the extreme outliers had an 18 ‐bpm difference between the 12‐L ECG and KM1L measurements, further supporting this affirmation.

Changes in HR between devices that led to a difference in numerical precision could be explained by a change in position from supine to sitting with an exaggerated response in relation to deconditioning, autonomic disturbances, as well as rapid changes in clinical status that can occur in ICU patients. Resting heart rate might be faster in a sitting position compared with lying, and variations in heart rate as well as QTc interval and dispersion can be produced by postural changes, especially in critically ill patients who may develop physical deconditioning and orthostatic intolerance (Aglawe et al., 2022; Borst et al., 1982; Fortrat et al., 1999; Jones et al., 2003; Levine et al., 1997; Mart et al., 2022; Nakagawa et al., 1999). Even though formulas try to correct QT according to heart rate, rapid changes in the R‐R interval secondary to position changes might not be adequately corrected by them (Kubo et al., 2005; Markendorf et al., 2018). Patients with acute cardiovascular illness (as 60% of our patients) are at higher risk of autonomic disfunction and consequently higher risk of ventricular arrhythmias (Bolton et al., 2007; Goldberger et al., 2019; Guo et al., 2021; Schmidt et al., 2001).

Our study included a critically ill population with various risk factors that could alter the QT interval. In our linear regression model, we found that some of these characteristics, including vasoactive drugs, potentially QT‐prolonging drugs, and antiarrhythmics, each resulted in a > 10 ms difference in QTc between devices and were present in 20%, 32%, and 33% of patients, respectively. We hypothesize that these medications could potentially lead to more extreme QTc values as well as swifter HR variations, accounting for less accurate measurements and corrections of the QT interval. As these conditions are more frequent in ICU patients than in general wards, they could also partially explain the difference found between numerical precision in our study compared with previous studies conducted in noncritically ill patients (Beitland et al., 2014; Tisdale et al., 2012). While the current analysis identifies these medications as contributing factors, further research may be needed to fully understand their individual and combined effects on QTc variability with different devices.

To our knowledge, this is the first study conducted in ICU patients evaluating the clinical validity of the mobile and readily available one lead ECG recorder KM1L. We had a well‐represented sample, with 60% of patients being admitted to the ICU for cardiovascular diseases and 20% having invasive mechanical ventilation, along with a high prevalence of QT‐prolonging risk factors. We conducted subgroup analysis as well as a regression model with findings that could partially explain differences in numerical precision in patients with certain medical conditions within the ICU.

Our study has certain limitations, including that it was conducted in a single center with only one KM1L device; therefore, no conclusions can be drawn regarding similar devices not evaluated in the present study.

Continuous cardiac monitoring with telemetry, though standard in many ICUs, is not universally available due to resource constraints, as in our study setting. Nevertheless, studies that have evaluated the correlation of telemetry monitors with real‐time QTc measurement have not shown a high correlation, particularly in patients with baseline ECG abnormalities, thus limiting its use as a replacement for 12‐L ECG (Nasser et al., 2021; Papukhyan et al., 2020). The number of patients with prolonged QT was low, with only 11% for males and 20% for females. However, prospective ICU studies have previously shown variable prevalences of acquired long QT in critically ill patients, ranging from 14% up to 61% in some studies (Hoogstraaten et al., 2014; Javanainen et al., 2019; Ng et al., 2004, 2008, 2010; Pickham et al., 2012; Tisdale et al., 2012). Our findings can only be interpreted in light of the population evaluated and may not be adequate to extrapolate our data to noncritically ill patients or patients with atrial fibrillation or active external electrical heart stimulation. We used Bazett's formula for QT heart rate correction, which has been previously shown to be less accurate than other formulas in certain situations different from our included population (Vandenberk et al., 2016). Nevertheless, it is the most commonly used correction formula and the one recommended for use in the Schwartz score for the diagnosis of prolonged QT syndrome, which must be actively screened for after drug cessation, as one‐fourth of patients with apparently acquired long QT syndrome harbor a pathogenic LQTS gene variant and may in fact have underlying congenital LQTS that is unmasked with QT‐prolonging drugs (Itoh et al., 2016; Krahn et al., 2022; Postema & Wilde, 2014; Vink et al., 2018).

## 5 CONCLUSION

Our results demonstrate that the use of a single‐lead KM1L is feasible and has good clinical precision in identifying patients with prolonged QT in the ICU. Caution must be held in the presence of certain medical conditions in critically ill patients that may render lower numerical precision.

## AUTHOR CONTRIBUTIONS

All authors contributed to the study conception and design. Material preparation, data collection, and analysis were performed by Martin Rebolledo‐Del Toro, Ana Beatriz Carvajalino‐Galeano, Clarena Pinto‐Brito, and Oscar Mauricio Muñoz‐Velandia. The first draft of the manuscript was written by Martin Rebolledo‐Del Toro, and all authors commented on previous versions of the manuscript. All authors read and approved the final manuscript and are to be accountable for all aspects of the work.

## FUNDING INFORMATION

We received no external funding in the development of this study.

## CONFLICT OF INTEREST STATEMENT

We, the authors, declare that we have no competing financial or nonfinancial interests that could be perceived as influencing the work presented in this manuscript. There are no relationships or activities that might have influenced or biased our work, and we have no financial or personal interests that could be construed as creating a conflict of interest regarding this manuscript.

## ETHICS APPROVAL AND CONSENT TO PARTICIPATE

We received ethical approval from the Local Ethics Committee (FM‐CIE‐0341‐22, 06/2022). The study was conducted following standards specified in the International Council for Harmonization Guidelines for Good Clinical Practice and in adherence to the principles of the Declaration of Helsinki.

## CONSENT FOR PUBLICATION

Not applicable.

## TAKE HOME MESSAGE

This study validates the clinical precision of KM1L, a single‐lead mobile ECG device, for identifying prolonged QT intervals in ICU patients. Caution is warranted in patients with certain medical conditions that may affect numerical precision.

## Supporting information


Table S1.

## Data Availability

Full study protocol as well as all raw data including 12‐L ECG and KM1L traces as well as statistical analysis will be available at request.
