# Supplementary material for: Use of portable single‐lead electrocardiogram device as an alternative for QTc monitoring in critically ill patients
Source: Ann Noninvasive Electrocardiol. 2024 Apr 16;29(3):e13116. doi: 10.1111/anec.13116 (PMC11021801; doi:10.1111/anec.13116)
Supplement: Supplementary file 1 — Table S1. [file ANEC-29-e13116-s001.docx]

**Supplementary Table 1. Backward stepwise multivariate lineal regression model.**

| **Source** | **SS** | **Df** | **MS** | **# observations**  **F(5, 124)**  **Prob > F**  **R-squared**  **Adj R-squared**  **Root MSE** | 130  6.04  0.0000  0.1958  0.1633  22.828 |
| --- | --- | --- | --- | --- | --- |
| Model | 15727.667 | 5 | 3145.533 |  |  |
| Residual | 64617.567 | 124 | 521.109 |  |  |
| **Total** | 80345.233 | 129 | 622.831 |  |  |

| **dif** | **Coef** | **Standard  error** | **T** | **P>\|t\|** | **95% CI** |
| --- | --- | --- | --- | --- | --- |
| Hipokalemia | -9.212 | 5.138 | -1.79 | 0.075 | -19.381; 0.957 |
| Vasoactive | -13.990 | 5.739 | -2.44 | 0.016 | -25.348; -2.631 |
| Antiarrhythmic | -12.873 | 4.974 | -2.59 | 0.011 | -22.720; -3.027 |
| QT prolonging drug | 13.840 | 5.411 | 2.56 | 0.012 | 3.130; 24.551 |
| HR difference ≥5bpm | -11.261 | 4.722 | -2.38 | 0.019 | -20.608; -1.916 |
| Cons | 6.601 | 2.826 | 2.34 | 0.021 | 1.008; 12.194 |
